# Study of the Impact of Operating Parameters and the Addition of Fat on the Physicochemical and Texture Properties of Extruded Snacks

**DOI:** 10.3390/foods14081307

**Published:** 2025-04-09

**Authors:** Nicolas Jacquet, Armande Plompteux, Yves Brostaux, Paul Malumba, Sabine Danthine, Christophe Blecker

**Affiliations:** 1 Gembloux Agro-Bio Tech, Food Technology Department, University of Liège, 5030 Gembloux, Belgium; plompteux.armande@gmail.com (A.P.); p.malumba@uliege.be (P.M.); sabine.danthine@uliege.be (S.D.); christophe.blecker@uliege.be (C.B.); 2 Gembloux Agro-Bio Tech, Modelling and Development Department, University of Liège, 5030 Gembloux, Belgium; y.brostaux@uliege.be

**Keywords:** extrusion, snack, starch, modeling, texture, response surface analysis

## Abstract

This study investigated the effects of extrusion parameters (barrel temperature, screw speed) and oil content on the physicochemical and textural properties of corn flour-based snacks, using a Box–Behnken response surface design. Significant predictive models (adjusted R^2^ > 90%) were established for specific mechanical energy (SME), expansion ratio, bulk density, hardness, compression work, water activity (aw) and dry matter content. The results showed that increasing oil content dramatically reduced SME (from 229.5 Wh/kg at 5% oil and 110 °C to 68.2 Wh/kg at 10% oil and 180 °C) and expansion ratio (maximum 3.73 at 145 °C, 150 rpm, 0% oil), while increasing bulk density (up to 0.271 g/cm^3^ at 10% oil). High oil content also led to a sharp increase in hardness (from 67.9 N at 0% oil to 466.9 N at 10% oil). Conversely, higher barrel temperature (up to 180 °C) and screw speed (up to 250 rpm) generally improved snack texture by reducing hardness and density and increasing expansion. Water activity ranged from 0.24 to 0.50 and was positively influenced by oil content and negatively by temperature and screw speed. Overall, oil content had the strongest detrimental impact on snack properties, but optimizing temperature and screw speed allowed the production of snacks with acceptable qualities.

## 1. Introduction

In our current society, changing lifestyles, cultural shifts and increasing sophistication have a profound impact on ways of eating. With limited free time, snacks have become increasingly popular [1,2] due to their convenience in consumption, preparation and storage, as well as their appearance and texture [3,4]. Among the technologies available for snack production, extrusion stands out as one of the most widely used processes due to its numerous advantages: it is fast, versatile, continuous, non-effluent and energy-efficient at low cost [5]. In addition, it enables the development of new products with unique shapes and sensory characteristics [6,7].

Extrusion is a physical process that transforms raw materials into various products of uniform shape and density under specific conditions of temperature, pressure, shear and moisture by forcing them to flow within a barrel and sheath and pass through a specifically designed orifice [8,9,10] to shape and/or expand them. This process is utilized in many food and non-food applications [9,11]. However, extrusion remains a complex process. Small variations in the parameters greatly affect the final quality of the product [12] through changes in its physicochemical properties [13,14], which play a major role in its consumer acceptability and functional properties [3]. The composition of the ingredients and their formulation also greatly influence the properties of snacks [3,15]. Typically, extruded food materials are made from starchy materials [16] due to their favorable expansion characteristics [2,17,18] resulting from high starch content [19].

During the extrusion process, the glass transition of starch plays a crucial role during the heating of the extrusion mixture and the cooling of the extrudates produced. Indeed, once the glass transition temperature (*T*_g_) is exceeded during heating, the molecular mobility increases. The amorphous phases become more malleable, transitioning from a glassy amorphous state to a rubbery state [20,21]. This secondary phase change facilitates the deformation of the mixture to be extruded. The glass transition can also occur at the exit of the extruder. Once in contact with the atmosphere, some of the water contained within the structure evaporates rapidly and cools the product [22,23].

The presence of water is also significant. Indeed, when heated in the presence of excess water, the starch granules absorb it, swell to maximum volume and finally burst, following the breaking of hydrogen bonds [6,24], dispersing their contents [25]. This irreversible phase transition allowing the conversion of starch to thermoplastic [26], which takes place after the glass transition and causes the collapse of molecular order, is called gelatinization [24]. This phenomenon is one of the most important in the extrusion of starchy food materials [27] as it contributes to the material being counted as a molten polymer [28].

In addition to starch and water, the presence of fat has a considerable impact on the functional and physicochemical properties of the product [29]. Indeed, during extrusion, the availability of amylose molecules increases [30] following the loss of the granule structure, which allows binding with lipids [31]. As a result, the formation of new crystalline structures, corresponding to amylose–lipid complexes, is favored. The formation of these starch–lipid complexes slows down the starch retrogradation [32]. Lipids are also responsible for many other reactions during extrusion: oxidation, destruction/formation of antioxidants and cis-trans isomerization of unsaturated fatty acids [33].

Besides composition, operating parameters also significantly influence the final properties of the extruded snacks. The temperature of the barrel generally increases expansion and decreases hardness and density [34]. Indeed, an increase in temperature increases the degradation of the material and the gelatinization of the starch, which in turn increases the amount of fragmented starch polymers melted into the extrusion mass [20]. This results in a drop in viscosity which encourages the growth of water bubbles in the form of superheated steam and allows the mass to expand rapidly [34]. Once at atmospheric pressure, these vapor bubbles become air bubbles that are trapped within the matrix, forming the expanded porous structure of the snacks. In addition, the increase in vapor pressure at higher temperatures leads to increased swelling, increasing the porosity; the higher the extrusion temperature, the more microporous the snacks are with more air cells [22]. On the other hand, when the temperature is excessively high, the trends described above are reversed: too high a temperature can result in a decrease in mass viscosity and an increase in vapor pressure within the melt, causing the bubbles to burst [34]. The temperature at which this transition occurs depends on the characteristics of the extruded ingredients. The screw speed exhibits a similar qualitative impact on snacks: it enhances expansion while reducing the density and hardness of the extruded products [34], but only when it is not excessively high [35]. The screw speed is responsible for the development of the shear rate and the average residence time of the mass within the apparatus [6]. A high screw speed results in a high shear force [22], which increases the degradation and gelatinization of the starch [36], and decreases the viscosity by increasing the elasticity of the mass [37]. Moreover, an elevated screw speed raises the absolute pressure inside the extruder: the pressure variation at the extruder outlet is therefore greater, resulting in greater expansion and increased porosity [22]. However, it is worth noting that a high screw speed significantly reduces the residence time within the extruder, potentially diminishing the energy received by the mass [5], thereby altering the trends described [34].

In recent studies, extrusion process optimization has focused on balancing texture, density and expansion in cereal-based snacks by adjusting formulation and process parameters. However, the combined impact of barrel temperature, screw speed and oil content on both textural and physicochemical characteristics remains insufficiently characterized.

## 2. Materials and Methods

### 2.1. Material and Sample Preparation

The maize flour used was obtained by grinding whole maize grains, supplied by Bauwens sprl (Sombreffe, Belgium), and the process was carried out at the faculty’s experimental farm (Gembloux, Belgium) using a hammer mill (Gladiator, Brussels, Belgium). The milling achieved a size of less than 1 mm. Composition of the flour is summarized in Table 1.

The oil used was Boni Selection unrefined cold-extracted rapeseed oil (Colruyt Group, Halle, Belgium).

The different recipes were prepared by sequentially adding the ingredients to an A 200 mixer (Hobart, Ivry-sur-Seine, France), capable of preparing mixes weighing 8 kg each. The ingredients (maize flour, water and oil) were mixed for 15 min using a flat beater-type agitator. Water and oil were added gradually while the mixer was running, and the order of incorporation remained constant for the production of all the samples (oil before water). The uniform dispersion of oil was indirectly verified by the consistency of process parameters and snack properties across replicates, as well as by the observed decrease in SME values with increasing oil content, indicating effective lubrication and even distribution.

### 2.2. Extrusion

**Figure 1:**
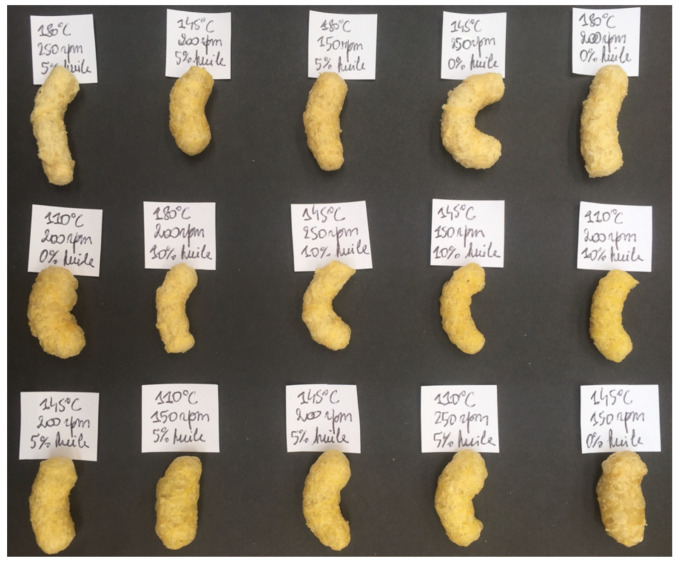
Visual appearance and morphology of extruded snacks produced under different operating conditions and oil contents.

The extrusion tests were produced using a BC 45 extruder–cooker (Creusot-Loire Clextral, Firminy, France) mounted with two 630 mm screws and 4 mm diameter round outlet dies. Parameter values (temperature and current intensity) were recorded continuously using a HERO6 Black camera (GoPro, San Mateo, CA, USA) and then transcribed every ten seconds. The visual appearance and morphology of each extruded snack produced under different operating conditions and oil contents are presented in Figure 1.

### 2.3. Specific Mechanical Energy (SME)

SME was determined as presented in several studies [8,9] according to the following equation:

$$SME\;[Wh/kg]=\frac{C\times v_{vis}\;}{Q}\times \frac{2\times \pi }{60}$$

where C is the torque of the three-phase electric motor supplying the screws [Nm], v_vis_ the screw speed [rpm] and Q the average mass flow rate [kg/h]. The torque of the three-phase motor was calculated by dividing the motor power by the screw speed:

$$Couple\;\left[Nm\right]=\frac{∆V\times I\times \sqrt{3}}{v_{vis}}$$

with ∆V being the voltage [V] supplying the extruder (220 V) and I the average current [A] passing through the extruder during the test (corrected by the no-load current).

### 2.4. Sample Characterization

The extruded snack samples were characterized in terms of their textural (expansion ratio, bulk density, hardness, crispness) and physicochemical properties (water activity, dry matter, water absorption (WAI) and solubility indexes (WSI) and crystallinity).

#### 2.4.1. Textural Properties

##### Expansion

The average expansion of the snacks was determined by a commonly used method [1,4] on 25 snacks via the expansion ratio, obtained by dividing the diameter of each of the samples obtained, previously measured using a 150 mm electronic digital caliper (RS Pro, Brussels, Belgium), by the diameter of the extrusion dies.

##### Bulk Density

The bulk density was calculated using the mass, diameter and length of the 25 extrudates according to the following equation considering the snacks as perfect cylinders [17,22]:

$$\rho_{b}=\frac{4\times m}{\pi \times d^{2}\times L}$$

where *ρ*_b_ is the bulk density [g/cm^3^], m is the mass of an extrudate [g], d is the diameter [cm] and L is the length [cm].

##### Hardness and Crispness

Hardness and crispness were assessed via a uni-axial bulk compression test using a Texture Analyser SME TA-XT2i Plus-Upgrade texturometer (Stable Micro Systems Ltd., Surrey, UK) equipped with a 50 kg load cell. An HD/P50 circular plastic extrusion probe (Stable Micro Systems Ltd., Surrey, UK) with a diameter of 45 mm was used to compress several snacks placed inside a plastic cell (internal diameter of 48 mm). The data were collected using three different thresholds (0.05, 0.3 and 1 N) and processed using the same software (Exponent version 6.1.16.0 (Stable Micro Systems Ltd., Surrey, UK)). The deformation curves obtained made it possible to characterize hardness and crispness.

Hardness was measured as the maximum force (peak force (N) applied by the flat circular probe during compression, as frequently proposed in the literature [38].

On the other hand, the crispness was evaluated according to the number of positive peaks (count peaks positive); this parameter is the most often used to determine crispness [39].

#### 2.4.2. Physicochemical Properties

##### Water Activity (a_w_)

Water activity was measured on samples using an AquaLab Series 3 TE (Decagon Devices Inc., Pullman, WA, USA) placed in a refrigerated cabinet at 20 ± 4 °C, the device having been calibrated using five known a_w_ standard solutions (a_w_ of 0.25 (13.41 mol/kg of LiCl in H_2_O)/0.50 (8.57 mol/kg of LiCl in H_2_O)/0.76 (6.00 mol/kg NaCl in H_2_O)/0.92 (2.33 mol/kg NaCl in H_2_O)) supplied by METER Group AG and distilled water (a_w_ of 1.00). Measurement was repeated five times.

##### Dry Matter (DM)

The DM of the snacks was determined by drying approximately 1 g of ground material in a ventilated oven (Memmert UF110) for 2 h 45 min at 130 °C, via the difference in mass before and after drying. Analysis was carried out in triplicate.

##### Water Absorption Index (WAI) and Water Solubility Index (WSI)

The water absorption index (WAI) and the water solubility index (WSI) were determined according to the following method, obtained after the adaptation and compilation of existing methods [1,38,40,41,42]: ±2.5 g (DM) of ground sample was weighed (m_gs_) and suspended in 25 mL of water at temperature in a centrifugation tube. Samples were shaken intermittently (5 min of shaking followed by 5 min of rest) for 1 h using a shaker (Fisher Scientific, Bioblock KL 2, Waltham MA, USA) then centrifuged at 3000× *g* for 20 min using a J-E Centrifuge Avanti^®^ centrifuge (Beckman Coulter Inc., Indianapolis, IN, USA) equipped with a JA-14 rotor. Supernatants were decanted and dried at 105 °C in a ventilated oven (Memmert UF110, Memmert GmbH, Schwabach, Germany) until a constant weight. The mass of the remaining gel in the centrifuge tube (m_gel_) and the mass of the dried residual solid from the supernatant (m_solide sup_) were noted. The WAI and WSI were therefore calculated as follows:

$$WAI\;[g/g]=\frac{m_{gel}}{m_{gs}}$$

$$SI\;\left[\%\right]=\frac{m_{solide\;sup}}{m_{gs}}\times 100$$

##### X-Ray Diffraction (XRD)

The crystallinity of the samples was evaluated in duplicate by XRD using a Bruker D8-Advance diffractometer (Bruker, Ettlingen, Germany) (λ Cu = 1.54 Å, 40 kV, 30 mA), using a Lynx- Eye (Bruker, Germany) and according to an established protocol [40,43]. Crushed samples were placed in a sample holder (depth of 0.8 and Z of 0.0122) and analyzed at ±20 °C on a 2θ range from 3° to 35°, with a step of 0.02° at a scanning speed of 1°/min. Calibration was carried out using corundum, silver behenate and stearin (β form) standards. The XRD patterns were analyzed (position and integration of the peaks) using the DIFFRAC.EVA V4.2 software. For all diffractograms obtained, a smooth factor of 0.230 was applied.

### 2.5. Response Surface Methodology (RSM)

A Box–Behnken response surface methodology (RSM) was created using Minitab 19.2020.1 software (Minitab LLC, State College, PA, USA). A fractional factorial was generated with three factors: two operating parameters (temperature and speed of the extrusion screws) and a formulation component (oil content). A Box–Behnken design was selected over a Central Composite Design (CCD) to avoid extreme factor combinations that could result in unstable extrusion conditions, while still allowing robust modeling.

Each factor had three normalized levels in the interval [1, −1] (see Table 2). Normalization (−1, 0, 1) was used to standardize factor levels, and quadratic terms were included to account for potential non-linear responses, particularly for oil content.

The dependent variables studied included the specific mechanical energy (SME), expansion ratio, bulk density, hardness, compression work, water activity (a_w_), dry matter (MS) and water absorption index (WAI).

Data were analyzed by multiple regression using the least squares method. For each of the dependent variables, a second-order polynomial Equation (1) was used to fit the experimental data to the independent variables.

(1) $$Y=\beta_{0}+\sum_{i=1}^{k}\beta_{i}x_{i}+\sum_{i=1}^{k}\beta_{ii}x_{i}^{2}+\sum_{i=1}^{k}\sum_{j=i+1}^{k}\beta_{ij}x_{i}x_{j}$$

where Y is the predicted response, k the number of independent variables (factors), x_i_ and x_j_ the coded independent variables, *β*_0_ a constant coefficient and *β*_i_, *β*_ii_ and *β*_ij_ the coefficients of the linear, quadratic and interaction terms.

The statistical significance of the terms in the regression equations was examined using analysis of variance (ANOVA) by calculating F-values and associated *p*-values (*p* > 0.05). The least significant terms were discarded in order to simplify, improve the fit and increase the accuracy of the model predictions.

## 3. Results and Discussion

### 3.1. Prediction Models

The average values of SME, texture and physicochemical properties of all samples as well as their standard deviations are summarized in Table 3 and Table 4. These data were utilized in the Box–Behnken experimental design to generate prediction models.

Response surface models were created by generating regression equations via Minitab. The non-significant terms (5%) were then disregarded to simplify the model, improve fit and enhance prediction accuracy. The adjusted and predicted R^2^ values were calculated to assess the model’s acceptability.

For seven of the dependent variables analyzed (SME, expansion ratio, bulk density, hardness, work, a_w_ and MS), a model deemed acceptable was generated by reducing the full model. Table 5 presents the coefficients and *p*-values associated with each of the terms, adjusted and expected coefficients of determination, and the goodness-of-fit test value for each of the polynomial regression equations, after term selection.

However, for the other dependent variables studied (the number of positive peaks, the average decrease, the linear distance, the WAI and WSI and the areas of the different peaks detected by X-ray diffraction), no sufficiently predictive (or even explanatory in certain cases) model could be established.

Regarding the significance of terms associated with the generated models, all linear terms in the models, representing the independent effect of each of the factors on the response variables, are significant; the means of the different modalities of the factors tested (x1, x2 and x3) are not all equal. This indicates that the three factors influence the seven dependent variables at a 95% confidence interval. Although these linear terms are generally very highly significant with *p*-values at 0.000, screw speed is only significant for bulk density (*p*-value of 0.019), and only highly significant for hardness and water activity (*p*-value of 0.001). These less pronounced significances reflect a greater probability that the correlations obtained for the aforementioned terms and variables are due to random variability.

For the quadratic terms (squared factor), screw speed was eliminated from all the models, given its very low statistical significance. The same applies to temperature, with the exception of the expansion ratio, which was retained. This implies that the relation between these factors and these variables did not show any significant curvature: the relationship is therefore linear. On the other hand, the quadratic term for oil content was retained in the equations, except for the work and dry matter variables. When retained, this term was significant in every case, except for hardness (*p*-value of 0.078). This leads to the conclusion that the relationships between oil content and the variables concerned follow a curved trend, and this at varying levels of certainty, ranging from an almost zero probability to a 7.8% chance (in the case of hardness) that this curved relationship is due to chance.

The presence and significance of the interaction terms, which express the effect of one factor depending on the values taken by another, fluctuate considerably depending on the response variable considered. As none of the interaction terms were retained in the EMS model, it can be concluded that interactions between factors do not play a part in the correlation established: the impact of each of the factors on the EMS is therefore independent of the other factors. For three of the seven variables considered, bulk density, hardness and work, the two oil content interaction terms (x1*x3 and x2*x3) were retained in the models and were at least highly significant: the relationship between these responses and temperature and screw speed depends on the oil content added to the mix. For the model relating to water activity, the two temperature interactions (x1*x2 and x1*x3) were retained, although they were not significant in view of the significance threshold set. The influence of temperature on screw speed and oil content for the response variable water activity is therefore relatively uncertain. A similar conclusion could be made from the *p*-values of the interaction terms of the dry matter model: although non-significant, the interaction terms contributing positively to the adjustment and predictive capacity of dry matter were retained. However, an interaction between the factors is not certain, especially for the interactions involving oil content (*p*-values of 0.280 and 0.163). Finally, regarding the expansion ratio model, all the interactions were retained and were all significant (maximum *p*-value of 0.021): the impact of each of the factors on the expansion of the snacks therefore depends on the other factors.

Considering the coefficients of determination, the results show, for all the models created, an adjusted R^2^ of at least 90%. Since the adjusted R^2^ is a measure of the closeness of the data to the regression line (adjusted to the number of terms in the model) and thus refers to the proportion of response variability attributed to the model rather than to random error, it appears that for these seven dependent variables, at least 90% of response variability is explained by the models. The models therefore fit the measured data very well. On the other hand, the predicted R^2^, which indicates the extent to which a regression model provides valid predictions for new observations, is also around 90%, except for the water activity model, where the predicted R^2^ is 77.2%. Although this last model is slightly less interesting in terms of prediction, it can nevertheless be concluded that the models generated should overall be sufficiently powerful to predict the properties of the snacks as a function of the temperature, screw speed and oil content conditions.

### 3.2. Impact of Operating Parameters and Oil Content on the Properties of Extruded Snacks

The impact of the three factors was examined separately, variable by variable, using Pareto charts and factorial diagrams. This is obviously only feasible for the properties of snacks whose models have been accepted. Pareto diagrams can be used to determine the value and importance of effects, which are presented normalized and in absolute values. To show the direction of the effects of the terms in the regression equations on the Pareto diagrams, the bars associated with terms that have a negative impact on the response variable have been hatched. Additionally, the vertical line indicates the limit of significance.

Factor diagrams are used to represent the relationships between responses and factors via the adjusted averages of responses. Two types were used in this study: main effect plots, representing the relationship between a response and the factors individually, and interaction plots, indicating the extent to which the relationship between a factor and an adjusted response depends on the value of a second factor.

**Figure 2:**
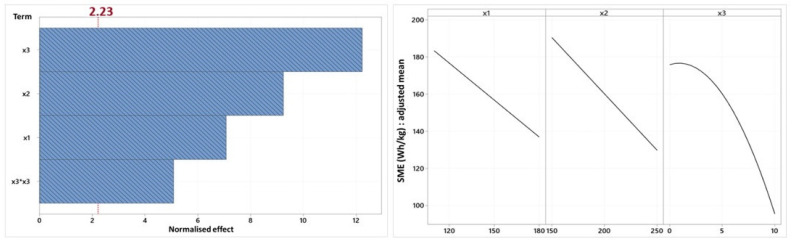
Pareto chart and factorial diagram of the main effects of the SME variable (x1: temperature, x2: screw speed (rpm), x3: oil content (%)).

Figure 2 shows the Pareto and the factor diagrams for SME. The term with the greatest influence on the SME variable is oil content, followed by screw speed and temperature. The position of temperature on the Pareto chart seems logical, firstly because it has already been observed that screw speed has a greater effect than temperature on SME [44] and secondly because some authors have not observed any impact of temperature on SME [40]. Regardless, all the terms retained in this model have a negative impact on this variable: an increase in any factor causes a decrease in SME and the relationship between oil content and SME follows a curved trend. These negative relationships with, on the one hand, temperature [24,45], and, on the other hand, oil content [46], are in accord with the literature. Increasing temperature helps to facilitate gelatinization of the starch, which is responsible for transforming the solid mass into a viscoelastic mass of low viscosity [36], and adding lipids helps to reduce macromolecular degradation. This reduction can be explained by the two roles of lipids explained above.

On the other hand, most authors state that the SME delivered by the extruder increases with screw speed [47,48,49], and justify this last statement by the presence of screw speed in the numerator of the EMS calculation. However, since the motor torque is also taken into account, the relationship between these two variables depends on the variation in the current required by the motor to rotate the screws. During the extrusion tests, it was observed that when the speed of the screws was reduced, the SME increased, as already proposed [24], because of the increase in current intensity. This can be explained by the fact that increasing the screw speed significantly reduces the residence time within the extruder, which in turn decreases the amount of energy transferred to the material [5]. It seems clear that the relationship between screw speed and SME is in this direction.

Finally, the main effects diagram clearly illustrates the presence and absence of quadratic terms for each of the factors: a decreasing linear relationship exists between SME and temperature and between SME and screw speed, while a decreasing curved relationship appears between SME and oil content.

**Figure 3:**
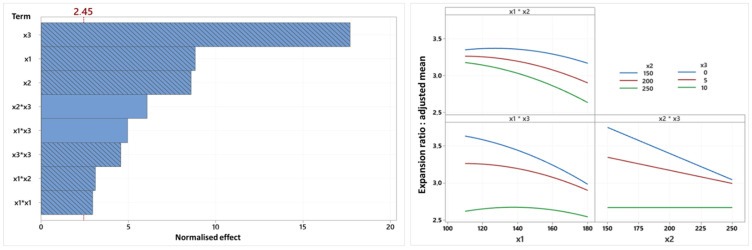
Pareto chart and factorial diagram of the interactions of the expansion ratio variable (x1: temperature, x2: screw speed (rpm), x3: oil content (%)).

Regarding the textural properties, Figure 3 presents the Pareto chart and factorial diagram associated with the expansion ratio variable. Overall, terms in the regression equation are negatively correlated with expansion, except for the two oil content interaction terms, which have a positive impact.

The linear terms have the greatest impact on this variable, with oil content having an effect that far exceeds the effects of the other terms, followed by the other terms associated with oil content (interaction and quadratic terms). On the other hand, for screw speed and temperature, opposite trends to those described previously, suggesting an increase in expansion with an increase in these variables, were observed [34]. However, similar trends were reported for the effect of temperature [22] and screw speed [19], respectively. There are several possible explanations for these results. First, the high temperatures, which cause greater degradation of the starch during extrusion, result in very low viscosity and excessive vapor pressure within the melt, which can prevent the bubbles from growing or even cause them to explode [22]. Secondly, given the fiber content (16.03%) and especially the insoluble fiber (13.15%) of the flour used, it can be assumed that fiber plays a role in the development of these particular expansion properties. In addition, a screw speed that is too fast considerably reduces the residence time of the mass [22], which does not receive sufficient energy to allow a correct gelatinization necessary for expansion.

The results also show that the relationship between the expansion ratio and temperature (x1*x3) as well as that between the expansion ratio and screw speed (x2*x3) depends on the oil content (and vice versa). The almost horizontal line observed on the two x3 interaction diagrams (x1*x3 and x2*x3) means that at 10% oil, expansion is virtually unaffected by changes in temperature or screw speed. This behavior may be due to the fact that at such a high level, the oil virtually prevents gelatinization of the starch through its lubricating effect, leading to a stable expansion that cannot be modified by the process parameters [33]. On the other hand, at 0 and 5% oil, expansion varies as a function of temperature and screw speed. The results show that the more drastic processing conditions lead to the biggest expansion decreases. The occurrence of these phenomena can again be explained by a reduction in gelatinization following the extreme conditions, and that the mass during extrusion does not have the capacity to react differently following a reduction in the residence time. In addition, high temperatures reduce the ability of lipids to bind with starch [25]. In addition, the relationship between the expansion ratio and temperature depends on the screw speed (x1*x2) (and vice versa): although the expansion ratio always decreases with increasing temperature for the three screw speed modalities, lower screw speeds still lead to greater expansion despite the increase in temperature, which is most certainly still linked to the residence time. Finally, it should be noted that the only factor with a linear relationship with the expansion ratio is screw speed, given the non-significance of its quadratic term, which was therefore eliminated from the model.

**Figure 4:**
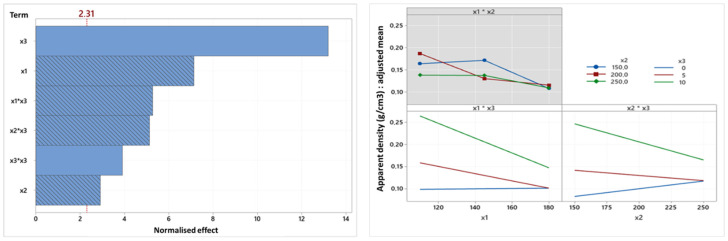
Pareto chart and factorial diagram of interactions for bulk density (N). (x1: temperature, x2: screw speed (rpm), x3: oil content (%)).

Always linked with the textural properties, Figure 4 shows the Pareto chart and factorial interaction diagram for bulk density, which is an important quality attribute of extruded snacks linked to the number and size of gas bubbles developing in the starch matrix during the process [2].

The diagram shows that two terms, the linear and quadratic terms of oil content, are positively correlated with bulk density, while all the others are negatively correlated. In addition, as per the SME and expansion ratio models, it is once again the linear oil content term that has the largest impact on bulk density. The effect of increasing bulk density with oil content is in line with the observation made earlier. While the three linear terms are generally found in the first positions of the Pareto diagram, the linear screw speed term is found here in the last position, close to the non-significance threshold, which means that screw speed is the term with the least influence on bulk density. The relationship between bulk density and temperature (x1*x3) and between bulk density and screw speed (x2*x3) depends on the oil content (and vice versa). When the oil content is 0%, the bulk density does not vary longer with the change in temperature. Conversely, when the oil content is 5 or 10%, the bulk density falls with the increase in temperature. These results highlight the indisputable impact of the oil content on the properties of the snacks. However, the impact of oil content on screw speed is different: at 0% oil, bulk density increases with screw speed, whereas it decreases at 5 or 10%. According to the literature sources consulted, this trend at 0% oil has never been described. Consequently, this aberrant behavior could be due to the low significance of the linear term of the screw speed (end of the x2 stick close to the red dotted line on the Pareto diagram) and that the blue line of the graph (x2*x3) should be rather constant according to the evolution of the screw speed.

**Figure 5:**
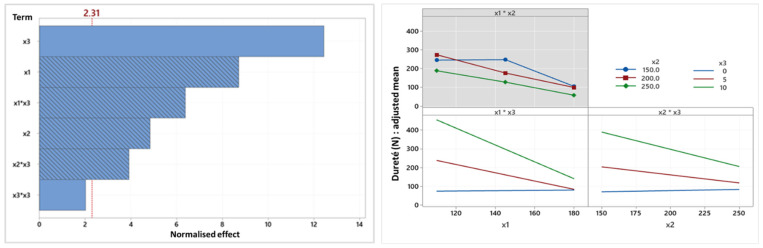
Pareto chart and factorial diagram of interactions of hardness (N) (x1: temperature, x2: screw speed (rpm), x3: oil content (%)).

Concerning hardness (Figure 5), the linear and quadratic terms of oil content are also positively correlated with snack hardness, while the other terms of the model are negatively correlated. These observations are in agreement with, on the one hand, observations concerning bulk density, and on the other hand, the literature showing that hardness is significantly impacted by temperature, screw speed and oil content [33].

The proportional relationship between hardness and oil content is again linked to its lubricating effect [33]. The negative correlation between hardness and temperature and between hardness and screw speed is also because high extrusion temperatures and screw speeds form snacks that are less dense and therefore less hard due to the thin walls that are formed [50] as a result of the low viscosity that develops within the extruder [38].

Further, the relationship between hardness and temperature (x1*x3) on the one hand and screw speed (x2*x3) on the other depends on the oil content: hardness remains constant whatever the temperature or screw speed for a 0% oil formulation, but falls as they increase for formulations with 5 and 10% oil. Although hardness is generally higher at higher oil contents, the adjusted mean hardness is, respectively, identical and virtually identical at oil contents of 0 and 5% for a temperature of 180 °C or a screw speed of 250 rpm. From this diagram, it is clear that the adjusted mean hardness varies as a function of oil content much more drastically at 110 °C and 150 rpm. These results are therefore still very much in line with those described for bulk density.

**Figure 6:**
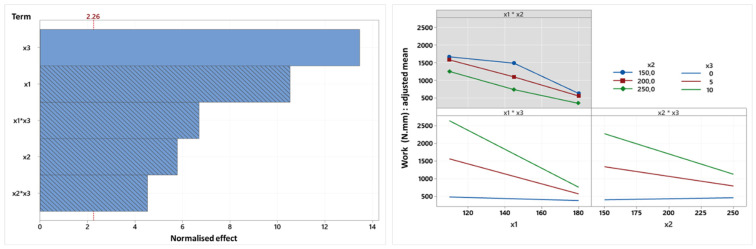
Pareto chart and factorial diagram of interactions relating to the variable compression work (N.mm) (x1: temperature, x2: screw speed (rpm), x3: oil content (%)).

Regarding the compression work, the Pareto diagrams for work (Figure 6) and hardness, which are very similar, have only one difference: the quadratic term for oil content, which is present in the hardness diagram (Figure 5), is not present in the work diagram. There is therefore only one term positively correlated with work, and the relationships are all linear. So, given the very strong correlation between hardness and work which is presented later, the conclusions deduced from the hardness diagrams can be extended to compression work, with the exception that greater differences in work exist between the oil content modalities at extreme temperatures (x1*x3) and speeds (x2*x3).

Finally, the last parameter studies were linked to the water properties. In order to fully describe the water status, the a_w_ and MS of the snacks were determined. Due to the high temperatures and pressures developed during the process, extrusion has an evident drying effect on the material: water evaporates from the mass once it is extruded from the dies under ambient conditions. Hence, both the a_w_ and MS of the mixes before extrusion are reduced in the extruded snacks.

**Figure 7:**
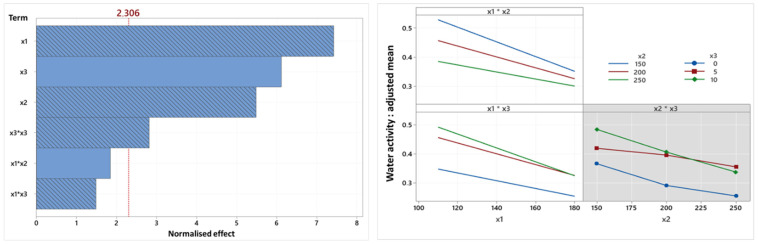
Pareto diagram and factorial diagram of interactions related to the water activity variable (x1: temperature, x2: screw speed (rpm), x3: oil content (%)).

**Figure 8:**
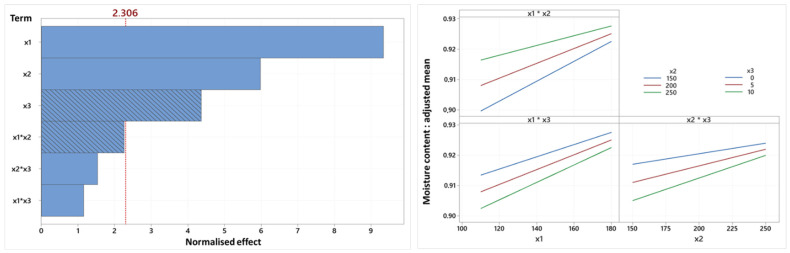
Pareto diagram and factorial diagram of interactions related to the dry matter variable (x1: temperature, x2: screw speed (rpm), x3: oil content (%)).

Figure 7 and Figure 8 present the Pareto diagram and the factorial diagram of interactions associated with the water activity and dry matter variables.

Regarding a_w_, similar to the temperature–screw speed interaction, the linear term of oil content is positively correlated with a_w_, while the other terms (linear terms of temperature and screw speed, quadratic term of oil content and temperature–oil content interaction) are negatively correlated. Moreover, the linear term of oil content, previously in the first position on the Pareto diagram, has been replaced by temperature. The impact of temperature on a_w_ is therefore more significant than oil content.

Considering that very few studies report the influence of operating or formulation parameters on a_w_, it has been difficult to formulate solid hypotheses regarding the positive correlation between a_w_ and oil content. However, bibliographic research suggests that oil, as a lubricant, reduces the shear exerted in the barrel, following the presence of surface fats on the granules, which (1) causes a lower pressure differential between the inside and outside of the extruder and (2) prevents the hydration of starch necessary for gelatinization. This pressure differential likely exerted a driving force for evaporation at the extrusion outlet, and the non-gelatinized starch has a low capacity to expand and reduces the mass’s extensibility, making it more compact. So, the residual water present in the material could be trapped inside the structure upon contact with the atmosphere, unable to escape. These two phenomena, unfavorable for water evaporation, contribute to obtaining snacks with higher water content and a_w_. Additionally, it is important to consider the water activity-depressing effect of starch: as previously discussed, a mixture containing oil proportionally contains less flour and therefore less starch, resulting in a lower reduction in a_w_. On the other hand, the negative impacts of temperature and screw speed on water activity were more evident: as temperature and screw speed increase, the temperature and shear rate of the mass increase. Consequently, starch degradation was more significant, favoring good water evaporation at the extruder outlet. The effect of screw speed was generally less pronounced than temperature effect on a_w_ and the dry matter of snacks.

Regardless of the screw speed and oil content, the relationship between water activity and temperature remains the same (x1x2 and x1x3). An increase in temperature linearly reduces water activity. However, there are nuances to mention. Firstly, there is a more intense difference in water activity between snacks produced at low temperatures compared to high temperatures for different screw speeds. This might be justified by the greater effect of the temperature factor compared to the screw speed factor on lowering water activity. Additionally, higher oil content leads to greater a_w_, but this difference is only slightly noticeable for formulations of 5% and 10% oil. Therefore, there may potentially be a threshold of fat content where all starch granules are surrounded by fat, beyond which neither the pressure differential, nor the hydration, nor the starch’s water activity-depressing effect are impacted by an increase in oil content. The occurrence of non-enzymatic browning reactions could possibly explain this phenomenon: at 5% oil, reactions such as Maillard reactions logically occur more, given the lesser lubricating effect of the oil, which allows better gelatinization and releasing relatively more water than at 10% oil. However, at 5% oil, evaporation is more favored than at 10% oil, leading to a more significant reduction in water activity. Therefore, the balance between these two phenomena could allow a_w_ to evolve in a roughly similar manner at 5% and 10% oil when the extrusion temperature is modified.

Regarding dry matter, Figure 7 shows that two of the six terms, oil content and the temperature–screw speed interaction, are positively correlated with this variable, while the other four terms (temperature, screw speed, screw speed–oil content and temperature–oil content interactions) are negatively correlated. Unlike other models, the linear terms of temperature and screw speed have the most significant impacts in the case of dry matter.

Obviously, as explained above for water activity, higher temperature and screw speed allow for better water evaporation, reducing the water content of snacks and thereby justifying the positive correlation between these variables and dry matter. However, oil prevents this evaporation. Therefore, the more the oil content increases, the more the proportion of dry matter decreases because snacks contain more water.

Our interpretation of the factorial diagram (Figure 8) allowed us to also conclude that the relationship between dry matter and the factors is always linear (due to the absence of a quadratic term in the model). Moreover, the diagram reveals that the slope of the linear regressions is contingent upon the modalities of the other factors. It also indicates that more severe processing conditions result in fewer variations in the impact of the factors on dry matter. This last observation could be explained by the fact that lower water content induces more difficulties to eliminate it through drying, as it is more bound.

Additionally, the formation of amylose–lipid complexes during extrusion likely contributes to the observed textural changes. These complexes may inhibit starch gelatinization and expansion by limiting the availability of free amylose chains, leading to reduced expansion ratios and increased hardness, particularly at higher oil contents. Although not directly assessed in this study, such complex formation could be confirmed in future work by combining Differential Scanning Calorimetry (DSC) and X-ray diffraction analyses to identify characteristic crystalline structures and thermal transitions associated with lipid–starch complexes. While water activity (aw) was mainly interpreted in terms of extrusion drying and process parameters, it is also likely that starch–lipid interactions contributed to the observed differences between samples. The formation of amylose–lipid complexes may immobilize water molecules within the matrix or influence microstructural properties, thereby affecting water retention and aw values. This effect could explain the higher aw observed in samples with greater oil content. Further studies using complementary techniques, such as DSC or sorption isotherm analysis, could help clarify the contribution of these molecular interactions to water activity variations.

With regard to starch gelatinization, the higher bulk density observed at lower barrel temperatures may be attributed to incomplete starch gelatinization due to an insufficient residence time in the extruder, which limits expansion and leads to denser structures with reduced porosity. This lack of sufficient gelatinization likely prevents the formation of stable vapor bubbles during extrusion, resulting in more compact products. Although not directly measured in this study, future work could include X-ray microtomography analysis to visualize internal morphology (pore distribution and wall thickness) and confirm the structural differences induced by varying extrusion temperatures and their correlation with gelatinization levels and bulk density.

The unexpected expansion behavior observed under certain extrusion conditions may also be partially attributed to the ingredient composition of the maize flour, which contains a high level of insoluble fibers (13.15%). These fibers can interfere with bubble formation by disrupting the starch matrix and limiting its elasticity. Furthermore, their ability to retain water may reduce starch hydration, consequently affecting gelatinization and expansion. Future studies should consider including a control formulation based on refined starch or low-fiber flour to better isolate and confirm the impact of fiber content on expansion properties.

The results of this study also highlight the complex interplay between SME and expansion. While SME decreased significantly with increasing oil content and higher barrel temperatures (reaching a minimum of 68.2 Wh/kg at 180 °C, 250 rpm and 10% oil), maximum expansion (3.73) was achieved under conditions of moderate temperature (145 °C), low screw speed (150 rpm) and absence of oil. These opposing trends suggest that achieving both minimal SME and maximal expansion simultaneously is challenging. Nevertheless, response surface analysis indicates that an intermediate set of conditions—characterized by moderate oil content (approximately 2–4%), elevated temperatures (around 160–170 °C) and screw speeds between 200 and 250 rpm—may offer a balance between process energy efficiency and product expansion. Further optimization studies using desirability analysis could help refine these target ranges to identify the ideal compromise between energy consumption and product quality.

## 4. Conclusions

The RSM, as intended, has enabled the establishment of robust predictive models (adjusted R^2^ between 90.3% and 97.5%) to anticipate the properties of extruded snacks based on the applied operating parameters and formulations. For the seven dependent variables analyzed (SME, expansion ratio, bulk density, hardness, work, water activity and dry matter), acceptable models could be generated. The results showed that SME decreased significantly with increasing oil content, from 229.5 Wh/kg at 5% oil and 110 °C to 68.2 Wh/kg at 10% oil and 180 °C. The expansion ratio reached a maximum of 3.73 at 145 °C, 150 rpm and 0% oil. Bulk density increased with oil content, up to 0.271 g/cm^3^ at 10% oil, and hardness rose sharply from 67.9 N (0% oil) to 466.9 N (10% oil). Water activity ranged from 0.24 to 0.50 depending on extrusion conditions and oil levels.

Three key conclusions emerged:

The expansion ratio did not follow the same trends as other textural properties. To better understand these mechanisms, future studies should consider characterizing snack porosity and internal structure, for instance through microtomographic imaging.

Screw speed, while having some influence, often had less impact than barrel temperature, highlighting the dominant role of thermal energy transfer.

No beneficial effect of oil addition on snack properties was observed within the tested range (0–10% oil). The strong negative impact on expansion and texture suggests that a narrower range (e.g., 2–6%) could be more appropriate; future research should investigate intermediate oil contents to determine thresholds where adverse effects begin.

Nevertheless, the results demonstrate that by modulating process parameters—such as increasing temperature up to 180 °C or adjusting screw speed to 250 rpm—acceptable product properties can be achieved, partially compensating for less favorable formulation choices. Finally, this study confirms that extrusion mastery is attainable through predictive modeling, offering a powerful tool for optimizing snack formulation and processing conditions. These findings fill an important gap in understanding how formulation and operating conditions can be combined to modulate snack quality, offering practical predictive tools for snack production optimization.

## Figures and Tables

**Table 1:** Chemical composition of the maize flour.

Components	Maize Flour	
	Average [% DM]	SD [% DM]
Starch	72.47	1.02
Total fiber	16.03	0.85
• Insoluble fiber	13.15	0.78
• Soluble fiber	2.88	0.32
Proteins	7.06	0.07
Fat	4.06	0.08
Ash	1.22	0.01
Total	100.85	
Water	13.17	0.02

**Table 2:** Box–Behnken response surface methodology.

Independent Factors	Encoded Levels		
	−1	0	1
Temperature [°C]	110	145	180
Screw speed [rpm]	150	200	250
Oil content [%]	0	5	10

**Table 3:** Response observed for SME and textural properties of snacks.

N°	Experimental Design Independent Variables			Secondary Extrusion Variable	Textural Properties										
							Texturometry								
	x1	x2	x3	SME [Wh/kg]	Expansion	Bulk Density [g/cm^3^]	Hardness [N]	Work [N.mm]	Count Peaks Positive			N Average Drop Off [N]			Linear Distance
									Thresholds: 0.05 N	Thresholds: 0.3 N	Thresholds: 0.1 N	Thresholds: 0.05 N	Thresholds: 0.3 N	Thresholds: 0.1 N	
	Temperature [°C]	Screw Speed [rpm]	Oil Content [%]												
1	180	250	5	106.5	2.59 ± 0.19	0.109 ± 0.019	58.4 ± 10.4	356 ± 60	302 ± 80	85 ± 39	15 ± 10	0.33 ± 0.08	0.88 ± 0.24	2.70 ± 1.30	279 ± 62
2	145	200	5	163.9	3.20 ± 0.11	0.122 ± 0.010	160.8 ± 27.0	988 ± 216	218 ± 74	104 ± 40	45 ± 13	1.51 ± 0.57	2.98 ± 1.09	5.97 ± 2.27	757 ± 134
3	180	150	5	172.1	3.14 ± 0.11	0.108 ± 0.008	105.7 ± 16.1	637 ± 132	261 ± 62	123 ± 32	50 ± 16	0.96 ± 0.27	1.87 ± 0.49	3.76 ± 0.81	597 ± 116
4	145	250	0	147.5	3.04 ± 0.09	0.115 ± 0.010	68.8 ± 12.5	397 ± 96	192 ± 45	88 ± 19	37 ± 11	0.92 ± 0.22	1.82 ± 0.42	3.55 ± 0.81	419 ± 80
5	180	200	0	155.7	3.03 ± 0.14	0.090 ± 0.009	67.9 ± 12.4	392 ± 96	177 ± 33	81 ± 17	33 ± 8	0.88 ± 0.22	1.73 ± 0.39	3.44 ± 0.68	383 ± 77
6	110	200	0	196.7	3.60 ± 0.18	0.102 ± 0.012	81.8 ± 11.4	518 ± 87	204 ± 18	124 ± 12	70 ± 9	1.89 ± 0.29	2.99 ± 0.41	4.91 ± 0.67	847 ± 116
7	180	200	10	68.2	2.56 ± 0.10	0.140 ± 0.011	131.9 ± 26.9	739 ± 185	249 ± 78	86 ± 38	25 ± 13	0.55 ± 0.21	1.35 ± 0.53	3.10 ± 1.25	402 ± 98
8	145	250	10	79.7	2.70 ± 0.12	0.159 ± 0.017	188.0 ± 27.3	1094 ± 226	233 ± 82	93 ± 36	36 ± 13	0.97 ± 0.44	2.15 ± 0.78	4.48 ± 1.46	600 ± 108
9	145	150	10	116.9	2.69 ± 0.09	0.251 ± 0.017	406.5 ± 60.1	2442 ± 488	277 ± 90	160 ± 69	70 ± 43	1.01 ± 0.36	1.68 ± 0.52	3.27 ± 1.14	957 ± 233
10	110	200	10	116.9	2.55 ± 0.13	0.271 ± 0.031	466.9 ± 67.2	2651 ± 783	303 ± 87	202 ± 63	95 ± 37	1.12 ± 0.30	1.59 ± 0.33	2.80 ± 0.57	1148 ± 214
11	145	200	5	155.7	3.12 ± 0.10	0.130 ± 0.010	173.4 ± 19.7	1096 ± 188	215 ± 75	107 ± 38	49 ± 12	1.48 ± 0.44	2.75 ± 0.74	5.15 ± 1.30	747 ± 103
12	110	150	5	229.5	3.40 ± 0.19	0.164 ± 0.020	245.7 ± 35.6	1670 ± 353	119 ± 49	69 ± 27	37 ± 8	2.35 ± 0.88	3.89 ± 1.34	6.40 ± 1.89	750 ± 129
13	145	200	5	147.5	3.17 ± 0.09	0.138 ± 0.012	196.7 ± 25.2	1226 ± 206	167 ± 69	86 ± 31	43 ± 15	1.54 ± 0.58	2.71 ± 0.84	4.70 ± 1.34	661 ± 132
14	110	250	5	145.3	3.22 ± 0.12	0.138 ± 0.012	189.0 ± 19.0	1258 ± 184	176 ± 53	94 ± 22	52 ± 15	2.22 ± 0.64	3.93 ± 0.95	6.55 ± 1.39	913 ± 114
15	145	150	0	203.4	3.73 ± 0.22	0.092 ± 0.013	90.1 ± 16.7	539 ± 127	225 ± 29	139 ± 17	79 ± 13	1.89 ± 0.39	2.96 ± 0.53	4.76 ± 0.86	931 ± 172

**Table 4:** Response observed for physicochemical properties of snacks.

N°	Experimental Design Independent Variables			Physicochemical Properties										
	x1	x2	x3	Water Activity	Dry Matter	WAI [g/g]	WSI [%]	X-Ray Diffraction: Peak Area at 2θ Angle:						
	Temperature [°C]	Screw Speed [rpm]	Oil Content [%]					±6.8°	±7.4°	±11.9°	±13.2°	±18.1°	±19.7°	±22.4°
1	180	250	5	0.32 ± 0.01	0.928 ± 0.002	3.69 ± 0.25	30.6 ± 5.5	0.497	0.000	0.346	0.953	1.111	0.000	0.000
2	145	200	5	0.37 ± 0.00	0.920 ± 0.002	4.25 ± 0.26	27.7 ± 3.1	0.491	0.000	0.549	0.905	1.705	0.000	0.633
3	180	150	5	0.34 ± 0.00	0.925 ± 0.001	4.27 ± 0.58	29.1 ± 6.3	0.656	0.000	0.550	0.911	2.268	0.000	0.000
4	145	250	0	0.26 ± 0.00	0.925 ± 0.001	3.53 ± 0.10	43.3 ± 1.3	0.442	0.000	0.376	0.815	1.252	0.000	0.000
5	180	200	0	0.24 ± 0.00	0.927 ± 0.000	3.13 ± 0.78	47.3 ± 9.4	0.503	0.000	0.349	0.964	1.211	0.000	0.000
6	110	200	0	0.35 ± 0.00	0.915 ± 0.001	4.12 ± 0.69	37.4 ± 8.1	0.769	0.000	0.551	1.216	2.209	0.000	0.000
7	180	200	10	0.31 ± 0.00	0.922 ± 0.001	4.08 ± 0.12	33.8 ± 3.4	0.336	0.000	0.237	0.428	1.062	0.000	0.000
8	145	250	10	0.34 ± 0.00	0.921 ± 0.000	3.49 ± 5.02	33.2 ± 2.3	0.376	0.000	0.271	0.549	1.010	0.000	0.000
9	145	150	10	0.48 ± 0.01	0.904 ± 0.001	5.02 ± 0.17	22.3 ± 0.6	0.000	0.000	0.000	0.929	0.646	2.547	0.561
10	110	200	10	0.50 ± 0.00	0.913 ± 0.000	4.57 ± 0.23	22.9 ± 1.4	0.000	0.239	0.000	1.479	0.310	2.430	0.705
11	145	200	5	0.41 ± 0.01	0.900 ± 0.001	4.26 ±0.10	28.3 ± 2.1	0.586	0.000	0.456	0.886	1.452	0.000	0.326
12	110	150	5	0.50 ± 0.00	0.913 ± 0.000	4.66 ± 0.08	22.7 ± 0.6	0.00	0.303	0.000	1.765	0.538	3.316	0.570
13	145	200	5	0.42 ± 0.00	0.915 ± 0.000	4.41 ± 0.09	28.5 ± 0.8	0.333	0.000	0.279	1.014	1.353	0.000	0.000
14	110	250	5	0.39 ± 0.00	0.916 ± 0.001	4.20 ± 0.13	27.0 ± 1.7	0.442	0.000	0.354	1.300	1.211	0.713	0.000
15	145	150	0	0.37 ± 0.00	0.945 ± 0.001	5.16 ± 0.05	24.4 ± 0.5	0.708	0.000	0.613	1.341	2.389	0.000	0.000

**Table 5:** Coefficients and *p*-values associated with each of the terms and determination coefficients of each model the asterisk (*) indicates non-significant terms).

Terms of the Model	SME [Wh/kg]		Expansion		Bulk Density [g/cm^3^]		Hardness [N]	
	Coefficients	*p*-Values	Coefficients	*p*-Values	Coefficients	*p*-Values	Coefficients	*p*-Values
Constant	393.5	0.000	3.128	0.000	524	0.000	1.07	0.000
x1	−0.6638	0.000	2.204 × 10^−3^	0.000	−1.43	0.000	−3.96 × 10^−3^	0.000
x2	−0.6070	0.000	4 × 10^−4^	0.000	0.58	0.000	−2.87 × 10^−3^	0.001
x3	1.74	0.000	−0.2783	0.000	738.3	0.000	4.06 × 10^−2^	0.000
x1^2^	-	-	−7.3 × 10^−5^	0.026	-	-	-	-
x2^2^	-	-	-	-	-	-	-	-
x3^2^	−0.977	0.000	−5.5 × 10^−3^	0.004	-	-	−1.449 × 10^−3^	0.078 (*)
x1*x2	-	-	−5.1 × 10^−5^	0.021	-	-	1.3 × 10^−6^	-
x1*x3	-	-	8.23 × 10^−4^	0.003	−2.552	0.000	−1.06 × 10^−4^	0.000
x2*x3	-	-	7.06 × 10^−4^	0.001	−1.206	0.001	-	0.004
R^2^ adjusted	95.64%		97.54%		95.49%		95.65%	
R^2^ predicted	92.67%		90.55%		89.17%		90.82%	
**Terms of the Model**	**Work [N.mm]**		**Water Activity**		**Dry Matter (%)**			
	**Coefficients**	***p*** **-values**	**Coefficients**	***p*** **-values**	**Coefficients**	***p*** **-values**		
Constant	524	0.000	1.07	0.000	0.829	0.000		
x1	−1.43	0.000	−3.96 × 10^−3^	0.000	5.35 × 10^−4^	0.000		
x2	0.58	0.000	−2.87 × 10^−3^	0.001	3.12 × 10^−4^	0.000		
x3	738.3	0.000	4.06 × 10^−2^	0.000	−3.63 × 10^−3^	0.002		
x1^2^	-	-	-	-	-	-		
x2^2^	-	-	-	-	-	-		
x3^2^	-	-	−1.449 × 10^−3^	0.023	-	-		
x1*x2	-	-	1.3 × 10^−6^	0.102 (*)	−2 × 10^−6^	0.054 (*)		
x1*x3	−2.552	0.000	−1.06 × 10^−4^	0.175 (*)	9 × 10^−6^	0.280 (*)		
x2*x3	−1.206	0.001	-	-	8 × 10^−6^	0.163 (*)		
R^2^ adjusted	96.50%		90.28%		91.18%			
R^2^ predicted	93.27%		77.20%		86.80%			

## Data Availability

The original contributions presented in this study are included in the article. Further inquiries can be directed to the corresponding author.
